# Remedies for Post-Partum Hemorrhage

**Published:** 1888-03

**Authors:** 


### REMEDIES FOR POST-PARTUM HEMORRHAGE.

### IPECACUANHA.

The above medicine and the disease for which it is named were given me by the Hahnemann Club, of Toronto, with the request that I would write on them an essay. In beginning I may remark that while all Hahnemannians believe in the totality of symptoms as furnishing the only guide for selection of the remedy, it is not so well known that it is not merely the *totality* that we require, but a *totality of characteristic symptoms*, for it is well understood that not unfrequently there will be a totality among two or three different remedies, and which then to choose with confidence is the knowledge which we most want.

As an illustration, and that my meaning may be better understood, let me refer to some, say two, remedies as confirmatory—for example, the œdema of Kali carb. and of Apis mel., both of which are very much alike. We have in each bag-like swellings above and below eyes, swellings of the face, hands, and feet, which by careful analysis will give us differences sufficient, perhaps, on which to prescribe; but if we possess some *characteristic conditions* of each remedy, the minor examination, which takes much time and is often a source of trouble to the busy practitioner, may frequently be avoided. The characteristics of the remedies referred to are, mainly, that *Kali carb. is a chilly remedy*, the patient, with very rare exceptions, seeking warmth, while that of *Apis mel. is usually the opposite*, almost every one under its influence desiring and seeking cold, cold rooms, fresh air, etc., the knowledge of such symptoms often enabling the practitioner to prescribe both rapidly and with accuracy—no small acquisition by our school. The object, then, of this paper will be very briefly to show, in *cases of emergency*, how a few prominent symptoms will often be of the greatest service, in the light of which conditions Ipecac. will be treated lastly.

CASE I.—I was called suddenly to see a Mrs. ——, a young, sanguine woman, threatened with miscarriage at the third month, and was no sooner in the room, than, observing that the pains were both frequent and strong, was ready to fear that the threatened loss was beyond my ability to arrest, and promptly making an examination, found, very much to my surprise, that the womb was perfectly intact, not apparently disturbed by the terrible pains which were going on. I waited awhile and then made another examination, the pains being severe, and during the latter of these I learned that the *abdominal walls were doing*

*all the work*, so that I had difficulty in preventing the uterus from being pushed through the vulva, which could only be prevented by very firm counter-pressure with the hand. Fortunately for my patient (who had all confidence), and also for her physician, I had been reading Hering's *Guiding Symptoms*, and there noticed under "Female Sexual Organs" of Amanita or Agaricus musc., "*Awfully bearing down pains, almost unbearable*," nothing being there said that they were *merely abdominal*, but knowing my patient as being *sanguine and very sensitive to cold*, prescribed on these two symptoms, giving one dose of Amanita$^{59m}$ (Fincke), which quickly and completely arrested the pains, the patient making a very rapid recovery. In this case one was called upon to act promptly; indeed, any failure would soon have taken her from my hands.

CASE II.—I was called by a professed homœopath to visit a lady who was in premature pains of the seventh month, and which could not be arrested. Being left alone with my patient, and supposing from the examination that her case was beyond the power of medicine, the os having enlarged and pains steady and severe, I had made up my mind for a night of it. While conversing with my patient I learned that she had suffered from a severe fright during the day. I at once thought that such a complication had better be out of the way, and gave her a dose of Opium$^{2c}$, when, to my surprise and pleasure, the Opium so removed the *cause* of her malady that the pains soon ceased, and I went home, she going her full term. Of course, all homœopaths know that we have no remedy so frequently indicated in recent frights as Opium, it being a characteristic symptom of this remedy, to know which, though only giving us a single characteristic, is to prescribe correctly.

This subject is voluminous and might be pursued *ad libitum*; one thing is certain, that the characteristic conditions of aggravation and amelioration should be the study of every physician, on the attainment of which no labor can be too great, where I may well mention a little work on this subject by Dr. E. J. Lee, well worth its weight in gold to all the busy men of our school.

Let me, then, proceed with the subject of this essay, Ipecac., which I will do very briefly in accordance with the cases named, wishing mainly that it shall be presented in its *salient* points, enabling every practitioner to give it without fear when needed. Bearing in mind the foregoing, let us look at Ipecac. in their light (*i. e.*, aggr. and amel.), which can also be best conveyed by illustration.

CASE III.—I attended Mrs. —— in labor, which went through naturally; the placenta discharged, the uterus contracted, and all apparently doing well. I was about leaving the house when my patient, a stout woman, exhibited a great and ominous paleness of the face, and very soon after this (almost immediately), nausea and vomiting; so, quickly placing my hand over the uterus, which not being found, I was led to look below, and found that the womb had suddenly expanded, while the vessels were pouring out *bright red blood* in profusion. I immediately inferred, from the terrible loss which was going on, that my patient could hardly live more than ten minutes unless she could be promptly relieved, and to call in aid at such a moment was impossible. I had then only two symptoms, but they were invaluable, *nausea and vomiting* and *severe loss of bright red blood*, on which I gave without delay, in pellets (for there was no time to dissolve any), Ipecac.$^{2c}$, repeating every five minutes. After the second dose the womb began again to contract and hemorrhage lessened; the third dose brought a full contraction with an entire cessation of the bleeding. I watched by the bedside a long time but finding no return of these symptoms, finally left, giving directions for good living to make up the loss (which I usually do the first two days), and my patient made a full and rapid recovery. I may add that, having attended this person in subsequent labors, the very same symptoms, threatening speedy loss of life, set in about half an hour after each delivery, and in the last case Ipecac.$^{1m}$ was used with the same success. Much may be said about this remedy; all the writer would now observe, is that whenever these two symptoms co-exist, *bright red blood* with *nausea and vomiting*, the hemorrhage will be controlled. Of course, I recommend the medicine high, having tried all. But some who write (so-called homœopathic books) do not know that our remedies manifest their curative effects almost in proportion as they may be triturated or succussed, being very wisely called *potencies*; but those who do not see these things should be docile and learn, for they are "hidden from the wise and prudent but revealed unto babes."

J. HALL, SR.

---

### BELLADONNA.

One of the leading indications for the use of Belladonna in hemorrhage occurring after labor is the presence of bearing down pains, with sensation as if the contents of the pelvic cavity would all be expelled. The blood is usually bright red in color and hot, coagulating rapidly after expulsion; sometimes it comes away dark and clotted and of a foul odor.

The flow is profuse and expulsion forcible, as a rule. The retention of the placenta is a decided indication for the use of this remedy. It appears to be specially serviceable in counteracting that condition of the uterus in which the contractive force, instead of being properly distributed over the body of the organ, is inclined to concentrate around the os and cervix, leaving the walls lax and the sinuses in a patulous state. We must be careful, however, not to depend entirely on the local symptoms. If we bear in mind the fact that we are treating the patient, rather than the disease, we will not be inclined to neglect the general indications for the use of the drug. These are, as a matter of course, much the same as in other cases, and include, among other symptoms, an exalted state of the mental faculties, sometimes amounting to mánia.

Frontal headache, with pressure and photophobia, flushed face, throbbing of the superficial vessels, and full, bounding pulse. These, and the other familiar symptoms usually calling for this remedy, will suggest its use in preference to any other medicine. We must constantly bear in mind the fact that parturition, *per se*, is a natural process, and if the patient were in a perfect state of health would go through its successive stages without any of these dangerous complications; consequently, when hemorrhage does take place to an extent to cause alarm, we have a state of disease to deal with, and in order to conduct our patient safely through the crisis, must search for and apply the simillimum.

It may be argued that such cases as those of placenta prævia are exceptions to the rule. We need only say, that if we have really to deal with purely local conditions, of course, local measures may be called for. But these cases are comparatively rare, and though requiring to be dealt with according to their individual needs, their occurrence does not affect the general rule. That mechanical measures are at times necessary must not be lost sight of, but it is still more important not to attempt to substitute these for dynamic ones when the latter are called for.

HAMILTON EVANS.

---

### CALCAREA.

While in most cases of post-partum hemorrhage calling for other remedies we are confronted with active *local symptoms* characteristic of the drug, in *Calcarea* cases there is such a dearth of these signs, we must look to the *history* of the patient, her *dyscrasia*, for, after all, it is the *dyscratic conditions* that *permit* such dangerous symptoms to arise.

If we are the family physician we are already cognizant of the facts that her menses have *usually* been *too early and too profuse* (though *scanty and regular* menses do *not by any means contra-indicate* Calc-carb.); that the least excitement brings on uterine hemorrhage; that her hands chap, and become sore and bleeding from working in water; that she is averse to open air and cannot stand cold, as it goes right through her, and that she is much troubled with cold, damp feet.

If called in for first time we will likely see that our patient has a general tendency to obesity, chest narrow, flabby, poorly developed muscles; she has fearful apprehensions of *death*, misery, and of sad events. Hemorrhage is *profuse and painless*, bright red, and is brought on or aggravated by slightest motion and least mental excitement. She has cold, damp feet; cold, damp legs, which are drawn up; chilliness, with desire for warmth and covering (contra, Secale); profuse perspiration, in bead-like drops, about *head* and shoulders, wets pillow all around head (Sil.); patient is usually better when lying in a horizontal position or head low, China (head raised). Hahnemann remarked, Calc. *peculiarly adapted* to persons whose pupils are *habitually* (in health) dilated. Finally, brethren, let us remember we are dealing with human beings, and not with mythological personalities yclept scarlatina, diphtheria, etc.

J. D. TYRRELL.

---

### CHINA.

We are led to think of the above remedy when the patient is of a *swarthy* skin, and the constitution broken down or debilitated by previous exhausting discharges.

Mentally, is low spirited, gloomy, has no desire to live.

*Intolerance of sensual impressions.*

Headaches are of an *intense, throbbing* character. Stitches from one temple to the other. The countenance is pale, livid, or hippocratic.

We find a longing for spirits, cooling things, *roasted coffee*—intense thirst for cold water, *a little at a time but often* (similar to Arsen. alb., but in the latter the water disturbs the stomach).

The abdomen is tympanitic, and is *not relieved by discharge of flatus.*

Darting, shooting pains, generally from right to left, *cross-wise in the hypogastric region.*

In uterine hemorrhages the flow is passive, blood dark, clotted, black.

In connection with such hemorrhage we find the patient fainting and convulsed. Surface of body and extremities cold. The mouth contorted, *fine* ringing in the ears, almost pulseless. She complains of scintillations or black motes before the eyes, or becomes blind.

Desires to be fanned—*gently* (Carbo veg., desire to be fanned *hard*).

In addition, as might be expected, she complains of giddiness, drowsiness, or loss of consciousness.

There are also *single jerks* in limbs and twitching and jerking of single muscles.

In atony of the uterus China frequently affords prompt relief—when labor pains cease from hemorrhage; *cannot have the hands touched.*

The sensitiveness of the patient is very great. She cannot bear the *slightest noise* or *excitement*, while the *slightest touch* aggravates the pain, which is relieved by a *firm, steady pressure.*

This *sensitiveness* of the whole nervous system is characteristic of this drug. In breathing a *puffing* noise is produced.

It is indicated for hemorrhage produced by Chamomile.

EDWARD ADAMS.

---

### CROCUS SATIVUS.

Great vacillation of mental mood, from cheerful hilarity to deep depression, or from tender affection to extreme anger, or *vice versa.*

The hemorrhage flows from the uterus in large dark or black clots and string-like masses, or forms into long, string-like masses and large clots as it flows from the vulva; is often offensive, *rarely bright red.*

Aggravated by the slightest motion.

Accompanied by bearing-down pain in back and groins, and sensation of something alive jumping around in abdomen, stomach, or chest. Extreme pallor of face; extremities cold as ice; pulse slow and feeble, or altogether pulseless.

Eyes fixed and shiny.

Foul odor from mouth.

Excessive thirst for cold drinks.

Oppression of chest, with desire to draw a long breath, as in yawning.

Sensation of coldness in back.

Sensation as if the hands had gone to sleep.

W. J. Hunter Emory.

---

### FERRUM.

When called to a case of labor it should be the duty of the accoucheur to recognize the constitutional state of his charge by careful inquiry relating to her prevailing conditions and ailments during pregnancy, etc., that he may be able to forecast the probability of a happy termination, or otherwise, of the impending event. Valuable indications relating to the selection of the most similar remedy may thus be disclosed to the careful observer before the denouement of post-partem trouble. He will thus, as it were, inform himself of the anamnesis of his case. The Ferrum patient is a cold patient. She presents an anæmic condition, marked by superficial congestion. Pseudo-plethora is the Ferrum keynote. The buccal cavity and tongue are pale and bloodless. She has, doubtless, throbbing in the blood-vessels, and the venous hum may usually be found.

Epistaxis of dark blood may also have been present at times; extreme paleness of face, which becomes red and flushed on the least motion, pain, or exertion. The mental state of Ferrum is characteristic; the least contradiction angers; her appetite is capricious, canine hunger alternating with anorexia; aversion to meat, which disagrees; aversion to eggs, fat or sour food; rest aggravates and continued motion relieves, though beginning to move causes aggravation; much worse at night, and especially after midnight.

Hemorrhage occurring in such a case will be characterized as follows: Copious discharge of partly fluid and partly black and coagulated blood, with pain in the loins and labor-like colic (in China the pains cease from the hemorrhage); in weakly persons, *chilly, with fiery-red face and thirst* (China, no thirst till after); vascular excitement, headache, and vertigo; constipation and hot urine; full, hard pulse and frequent short shuddering.

Ferrum has spasmodic labor pains and dryness of vagina, and is of service in excessive and prolonged labors, and is also adapted to states consequent upon severe and copious hemorrhages marked by pale face of bloated appearance; skin cold, pitting on pressure, especially about the ankles; great lassitude, tearfulness, constipation.

